# Compliance with the smoke-free public places legislation in Nepal: A cross-sectional study from Biratnagar Metropolitan City

**DOI:** 10.1371/journal.pone.0264895

**Published:** 2022-03-09

**Authors:** Lila Bahadur Basnet, Shyam Sundar Budhathoki, Biplov Adhikari, Jeevan Thapa, Bandana Neupane, Talibita Moses, Meghnath Dhimal, Paras K. Pokharel, Anup Ghimire, Deepak Belbase, Sudip Khatri, Narendra Kumar Yadav, Richard J. Pinder

**Affiliations:** 1 School of Public Health and Community Medicine, B.P. Koirala Institute of Health Sciences, Dharan, Nepal; 2 Department of Primary Care and Public Health, School of Public Health, Imperial College London, London, United Kingdom; 3 Mulpani Primary Health Center, Ministry of Health and Population, Kathmandu, Nepal; 4 Department of Community Health Sciences, Patan Academy of Health Sciences, Lalitpur, Nepal; 5 Nepal Health Sector Support Programme (NHSSP)/DFID, Government of Nepal Ministry of Health and Population, Kathmandu, Nepal; 6 School of Sciences, Nkumba University, Kampala, Uganda; 7 Research Section, Nepal Health Research Council (NHRC), Ramshah Path, Kathmandu; Tribhuvan University, Peoples’ Dental College, NEPAL

## Abstract

**Background:**

Smoke-free legislation banning tobacco smoking in public places was implemented across Nepal in 2014 with the ambition to reduce the impact of second-hand smoking. As part of a comprehensive policy package on tobacco control, the implementation of the legislation has seen a marked reduction in tobacco consumption. Yet there remains uncertainty about the level of compliance with smoke-free public places.

**Objectives:**

This study assesses the compliance with smoke-free laws in public places and the factors associated with active smoking in public places in Biratnagar Metropolitan City, Nepal.

**Methods:**

A cross-sectional study was conducted in the Biratnagar metropolitan city in Province 1 of Nepal from July to December 2019. A total of 725 public places within the metropolitan city were surveyed using a structured survey tool. Active smoking was the primary outcome of the study which was defined as smoking by any person during the data collection time at the designated public place.

**Results:**

The overall compliance with smoke-free legislation was 56.4%. The highest compliance (75.0%) was observed in Government office buildings. The lowest compliance was observed in eateries, entertainment, and shopping venues (26.3%). There was a statistically significant association between active smoking and the presence of ‘no smoking’ notices appended at the entrance and the odds of active smoking in eateries, entertainment, hospitality, shopping venues, transportations and transits was higher compared to education and health care institutions. None of the ‘no smoking’ notices displayed fully adhered to the contents as prescribed by the law.

**Conclusion:**

As more than half of the public places complied with the requirements of the legislation, there was satisfactory overall compliance with the smoke-free public places law in this study. The public venues (eateries, shopping venues and transportations) that are more frequently visited and have a high turnover of the public have lower compliance with the legislation. The content of the message in the ‘no smoking’ notices needs close attention to adhere to the legal requirements.

## Introduction

Tobacco smoking is as ancient as the human civilization itself. Despite widespread knowledge of its harmful consequences on human health, smoking remains highly prevalent. It not only endangers the health of the smoker but also of those exposed to the fumes involuntarily through second-hand tobacco smoke (SHS) or environmental tobacco smoke. The Institute of Health Metrics and Evaluation estimated that 2% of the deaths worldwide were attributed to second hand smoke alone in 2017 [1]. Almost one-third of the adult population is exposed to SHS worldwide [2] with 1.2 million premature deaths annually in non-smokers attributed to SHS in 2021 [3]. Half of the world’s children are exposed to SHS in public places with an estimated 65,000 deaths annually sue to SHS [3]. Short-term exposure to SHS places an individual at approximately 80% to 90% of the cardiovascular risk to that of first-hand smoking [4]. Of concern is the risk associated with women and children who are exposed.

In Nepal, it is estimated that tobacco kills about 27,000 people every year [5]. Tobacco is attributed to 14.9% of all deaths and 28.0% of deaths due to cardiovascular disease [6]. The Noncommunicable Disease Risk Factors STEPS survey of 2019 reported 28.0% of men and 7.5% of women smoke tobacco. Exposure to secondhand smoke has been reported among 22.5% of adults in 2019 which is a slight decrease from 37.2% in 2013 [6]. The World Health Organization Framework Convention on Tobacco Control (WHO FCTC), one of the most quickly ratified treaties in the history of the United Nations, is an agreement to counter the global tobacco epidemic and protect people against the harmful effects of smoking [7]. Nepal ratified the WHO Framework Convention on Tobacco Control (FCTC) in 2006 [8]. Nepal’s Tobacco Product (Control and Regulatory) Act, 2011, governs tobacco control, regulates smoking in public places, tobacco advertising, promotion and sponsorship, and tobacco packaging and labelling. More recently the Tobacco Product Control and Regulatory Directive 2014 was implemented, mandating the smoke-free public places policy nationwide. Nepal has also adopted the MPOWER measures in curbing the use of tobacco as outlined in the WHO FCTC [9]. By launching the ‘FCTC strategy 2030’ in 2018, Nepal has made further commitments towards the enforcement of smoke-free laws [10].

The Tobacco Product (Control and Regulatory) Act, 2011, mandates 100% smoke-free public places to prevent SHS exposure; exceptions include airports, hotel/lodgings, and prisons (smoking areas are designated in these places). In 2017, the WHO South-east Asia regional office-led review of the implementation of MPOWER measures among the countries in the region lauded Nepal for huge progress in terms of legal provisions for tobacco control in Nepal [10]. With constant interference from the tobacco industry, complete and absolute implementation of the act seems to be more difficult than ever. Several studies [11–13], including an editorial in *The BMJ* [14] stated international investment in tobacco control is more important than ever as low and middle-income countries have limited capacity to withstand the tobacco industry lobbying against tobacco control measures. There has been consistent interference by the tobacco industry since the time of enactment of the legislation in Nepal [8], [15].

Yet very little is known regarding compliance with smoke-free laws in Nepal which are intended to prevent second-hand smoke exposure in public places. Evidence from other countries shows that compliance surveys provide longitudinal means of measuring the progress of the smoke-free legislative impact [16, 17].

With limited monitoring and enforcement guidelines available for tobacco control, there is a dearth of research evidence available to report the effectiveness of the tobacco control legislation in Nepal. As Nepal advocates for healthy cities, smoke-free public places are the cornerstones of that goal. There is a need for field-based reports to better understand the baseline implementation status and provide evidence to national and municipal authorities on policy and other implementation options to realise smoke-free cities. With this background, we intend to present compliance of the ‘smoke-free public places’ legislation in a large metropolitan city from Province 1 of Nepal. The evidence generated from this study is expected to provide baseline data for the city being studied and a case study for other major cities including Kathmandu, the capital city to initiate an evidence-based assessment of the smoke-free policy implementation at the local level. Therefore, this study aims to assess the implementation status of smoke-free legislation in Biratnagar metropolitan city in province 1 of Nepal.

## Materials and methods

### Study design

This is a cross-sectional study conducted in Biratnagar, a metropolitan city in eastern Nepal with data collection undertaken between July and December 2019. Biratnagar was chosen for the study as the closest Metropolitan city to the host organization (BP Koirala Institute of Health Sciences) for this research [18]. This study was conducted in 2019, which is 7 years after the enactment of the Tobacco Product (Control and Regulatory) Act in 2011 [19] and 5 years after the initiation of the policy implementation soon after the release of the Tobacco Product Control and Regulatory Directive in 2014 [20].

### Sample size

As we did not find any prior research reporting compliance of the smoke-free policy in Nepal, we calculated the sample size for the study based on a study conducted by Goel et. al. in Punjab, India [21]. The study reported 83.8% overall compliance with the smoke-free legislation in public places. From the study, we took the non-compliance proportion of the public place with the highest non-compliance of 21.2% in the transits/transportations for our sample size calculation to maximize the sample size. We considered a power of 85% and added 10% for adjusting for non-response in our sample size which came out to be 720 public places.

### Study tool

The data collection tool was adapted from the guide on ‘Assessing compliance with smoke-free law’ developed as a part of a collaborative effort between the Campaign for Tobacco-Free Kids, Johns Hopkins Bloomberg School of Public Health and International Union against Tuberculosis and Lung Disease (IUALTD), 2014 [22]. The tool was developed for WHO by the IUALTD/Johns Hopkins as a valid tool for assessing the compliance of smoke-free laws. The tool includes an observational checklist for assessing public places which can be assessed in S1 File.

### Study settings and data collection

Biratnagar Metropolitan City is in the Morang district of Province 1. It is the largest city of Province 1 and the second-largest city in Nepal. It occupies an area of approximately 77 km^2^, divided into 19 wards as administrative units. The population of the city is estimated at 218,526 [23]. The city contains 19.8% of the total population of Morang District [24].

In coordination with the Metropolitan City office of Biratnagar, the public places which are accessed more by the public were chosen purposively as the list of public places was not available and the city map did not populate the public places in it. In the process, the city centre as identified by the Metropolitan office was taken. Public places in all directions of the main city centre were visited. All consecutive public places were considered for the study as they were encountered and care was taken to visit all types of public places as categorized in the Tobacco Products (Control and Regulation) Act, 2011 of Nepal [19]. The selection of study units was limited to the public venues under the jurisdiction of the Biratnagar metropolitan office.

Two researchers surveyed the public places and carried out the compliance observations (once per public place) at unannounced timings (between the office/peak hours) to seek typical behaviours. The two researchers were trained using the training instructions available on the guide on ‘Assessing compliance with smoke-free law’ [22], which included reviewing the requirements of the law, orientation with the data collection forms and the protocol, pilot data collection along with Principal Investigators (SSB and LBB) in Dharan (another city in east Nepal), and debrief after pilot data collection to share experiences and harmonize the findings. The PIs also visited 70 public places on random days (~10% of the sample size) during data collection as a measure of quality control to ensure consistency of results. During the data collection for the study, all collected forms were checked for completeness and inconsistencies for data on the same day by the PIs. The researchers visited workplaces (including factories) and government buildings, healthcare institutions, and educational institutions during office hours (09:00–17:00), hospital visiting hours (10:00–11:00 and 17:00–18:00), and school hours (10:00–16:00), respectively. The researchers surveyed eateries, shopping venues, entertainment centres, transit sites, and public vehicles during their busiest hours (17:00–20:00). Depending on the area, each site was surveyed for around 20–30 minutes. The possible repeated observations of public transportations (buses and minibuses) were avoided by noting the vehicle numbers and excluding the already observed ones. The law has designated smoking areas for smoking preferably outside or within the building with smoke exiting directly to the outside at prisons, airports and hotels. The study excluded prison from our study as it required special permission and security clearances. For airports and hotels, the researchers observed outside the buildings to check for smoking outside the designated areas.

### Variables

Compliance with the smoke-free law in this study was measured based on the 8 variables listed in the structured observational checklist for all 6 categories of public places. The variable was recorded as present/absent based on observation. The main outcome variable denoting compliance was active smoking in a public place. Secondary variables for compliance were, ‘no smoking’ notice, ‘no smoking’ notice at the entrance, size of the ‘no smoking’ notice compliant with the law, cigarette smell, cigarettes/bidi stubs/butts, smoking aids (ashtrays, matchboxes, and lighters), and active smoking by owner/manager/staff. Total compliance for each type of public place was reported by calculating the average percentage of each variable.

Active smoking in a public place: Active smoking in a public place was marked as present if anyone was seen smoking during the researcher’s visit at the public place being observed for the study.

‘No smoking’ notice: In public venues where smoking was completely banned, if a notice along the lines of, “smoking and consumption of tobacco is prohibited in this area and/or is injurious to health,” was displayed in the public place including any written message to prohibit smoking (and/or lawful repercussions), a ‘no smoking’ notice was marked as present [20]. In public venues where the legislation allowed the manager to designate smoking area (tourist hotels and airport), we looked for ‘no smoking’ notice to be along the lines of “smoking and consumption of tobacco is prohibited in this area and/or is injurious to health,” combined with “smoking or consuming tobacco in areas other than designated is punishable by law” [20].

‘No smoking’ notice at the entrance: ‘No smoking’ notice at the entrance was marked as present if the public place appended the notice at the main entrance of the venue.

‘No Smoking’ notice of size compliant with the law: The size of the appended notice compliant with the law was marked as present, if the ‘no smoking’ notice displayed was at least 30cm x 20cm at the main entrance and 20cm x 15cm in the inner door of the public place building as per the law in Nepal [20]. For this study, compliance was marked present only if the size at both places were correct.

Cigarette smell: The cigarette smell was marked present if the researcher could detect any tobacco smoke smell during their visit.

Cigarette/bidi stubs/butts: Cigarette/bidi stubs/butts were marked present if any such items were detected within the public places during the researchers’ visit.

Smoking aids: Smoking aids was marked present if any of the items used to light the cigarette, collect ashes or dispose the cigarette/bidi stubs/butts was present in the venue. These items are provided by the venue manager or staff.

Active smoking by the owner/manager/staff: Active smoking by the owner/manager/staff was marked as present if the active smokers noted by the researcher was identified as owner, manager, or staff. The researchers first noted any smoking as they entered the venue. The person smoking was later classified as owner/manager/staff or general public based on identification either by uniform or during introduction/consent for the study or by direct confirmation towards the end of the study. Care was taken not to note any details other than their status as owner/manager/staff. Co-operation was received in all venues when asked about their status as owner/manager/staff.

The Tobacco (Control and Regulation) Act 2011 [19], and the Tobacco Product Control and regulatory Directive 2014 [20] define public places as “places accessed by the general public, built by the government, private companies or individuals for public or group activities.” This includes government offices, educational institutions, libraries, training institutions, health-related institutions, airports, airlines, public transportations, child welfare centres, child care centres, orphanages, old age homes, children parks, children clubs, public latrines, workplaces (indoor/outdoor), cinema halls, cultural centres, theatres, hotels, resorts, restaurants, bars, dining halls, canteens, lodges, hostels, guest houses, stadiums, pool houses, departmental stores, pilgrimage, religious places, public vehicle waiting spaces, and ticket counters.

Public places, as per the law in Nepal, were categorized into 6 groups, 1) eateries (including restaurants, bars, and pubs), hospitality, entertainment, and shopping venues, 2) educational institutions, 3) public transportations (bus and minibus) and transits (including public toilets), 4) government office buildings, 5) healthcare facilities, and 6) workplaces (indoor/outdoor spaces of factories, private offices, and small industries).

For the univariable and multivariable analysis, the presence of active smoking was considered as the dependent variable, while other variables were considered as the covariates. For facilitating the multivariable analysis, the public places were also recategorized into three categories by merging:

1. Eateries, entertainment, hospitality and shopping venues with public transportations, and transits: This was done keeping in mind that both these venues have a high turnover of people per day and are managed by a venue manager/staff who provide retail service to people in return for a fee for service or goods.
2. Educational institutions with healthcare facilities: This was done keeping in mind that both these venues have a more structured schedule and regulations for the public who are either students, teachers, patients, or doctors who have come to these venues to get a specific service. These deal with special and/or vulnerable subgroups of the public.
3. Government office buildings with workplaces: This was done keeping in mind that both these venues are mostly for employed workers with some visits by the general public for services. These venues have regulations set by employing organizations.

#### Ethics

Ethical approval for the study was obtained from the Institutional Review Committee, B.P. Koirala Institute of Health Sciences, Dharan, Nepal (Ref. No: 189/075/076-IRC). We also sought written permission and cooperation from the office of Biratnagar Metropolitan City. We took informed consent and shared the purpose of the study with the owner/manager of the public places studied and assured the confidentiality and anonymity of the person and places.

### Statistical analysis

The data collected each day were checked for completeness and entered into a spreadsheet at the end of each day. The data were then cleaned, coded, and exported to IBM Statistical Package for the Social Sciences (SPSS) version 22 for analysis. Cross-tabulation of active smoking with its predictors was done reporting the counts and the percentages. Univariable logistic regression was applied to assess the relationship of each predictor with active smoking generating crude odds ratio and the level of significance (p-value). All the variables which were significant at p<0.1 were considered for multivariable analysis. Collinearity was tested among these variables by calculating the Variance Inflation Factor (VIF), and the variables with VIF < 2 were included into the multivariable logistic analysis.

## Results

We collected data from a total of 725 public places for compliance with the smoke-free legislation. Out of the 725 public places, 503 (69.3%) were eateries, entertainment, hospitality and shopping venues, 87 (12.0%) educational institutions, 52 (7.0%) public transportation and transits, 28 (3.8%) government office buildings, 16 (2.2%) healthcare facilities, and 41 (5.6%) workplaces.

We observed the average population compliance with smoke-free laws across the metropolitan city to be 56.4%. Government office buildings had the highest compliance of 75.0%. The lowest compliance was observed among eateries, entertainment, hospitality and shopping venues at 26.3%. The absence of observed active smoking in public places ranged from 38.5% in public transportations and transits to 98.9% in educational institutions.

Only 1.0% (5/503) of eateries, entertainment, hospitality, and shopping venues had appended a ‘no smoking’ notice, whereas all the government office buildings appended the notice. The size of the appended notice was compliant with the law in 1.0% (5/503) of eateries, entertainment, hospitality, and shopping venues and 62.5% (10/16) of healthcare facilities.

The smell of tobacco smoke was noted in 25.2% (127/503) of eateries, entertainment, hospitality, and shopping venues, whereas no such smell was noted in educational institutions. Cigarettes/bidi stubs were absent in 23.5% (118/503) of eateries, entertainment, hospitality and shopping venues and 97.7% (85/87) of educational institutions. Smoking aids were absent in only 90.4% (47/52) of public transportations, and transits and 24.5% (123/503) of eateries, entertainment, hospitality and shopping venues. All other places were completely devoid of smoking aids.

Among owners/managers/staff of educational institutions, 98.9% (86/87) were not found actively smoking, whereas only 36.5% (19/52) of owners/managers/staff of public transportations and transits were not found to be actively smoking (Table 1).

**Table 1:** Compliance with specific indicators of smoke-free legislation at different public places in the Biratnagar metropolitan city (%).

Type of public place.	Eateries, entertainment, hospitality and shopping venues n = 503 (%)	Educational Institutions n = 87 (%)	Public transportations and transits n = 52 (%)	Government office buildings n = 26 (%)	Healthcare facilities n = 16 (%)	Workplaces n = 41 (%)
**Absence of active smoking in a public place**	233(46.3)	86(98.9%)	20 (38.5)	22 (84.6)	11 (68.8)	28 (68.3)
**Presence of ‘no smoking’ notice**	5 (1.0)	29 (33.3)	38 (73.1)	26 (100)	10 (62.5)	9 (22.0)
**Presence of ‘no smoking’ notice at the entrance**	2 (0.4)	14 (16.1))	17 (32.7)	12 (46.2)	10 (62.5)	4 (9.8)
**‘No smoking’ notice size compliant with the law**	5 (1.0)	17 (19.5)	6 (11.5)	12 (46.2)	10 (62.5)	9(22.0)
**Absence of cigarette smell**	127 (25.2)	87 (100)	16 (30.8)	22 (84.6)	13 (81.3)	37 (90.2)
**Absence of cigarettes/bidi stubs/butts**	118 (23.5)	85 (97.7)	16 (30.8)	14 (53.8)	11(68.8)	11 (26.8)
**Absence of smoking aids**	123 (24.5)	87 (100)	47 (90.4)	26 (100)	16(100)	41 (100)
**Absence of active smoking by owner/manager/staff**	443 (88.1)	86 (98.9)	19 (36.5)	22 (84.6)	11(68.8)	30 (73.2)
**Overall Compliance (%)**	26.3	70.6	43.0	75.0	71.9	51.5

Total average compliance = 56.4%

Statistically significant associations (p-value <0.001) were found between active smoking and all the predictors of smoking behaviour in bivariate analysis (Table 2).

**Table 2:** Predictors of smoking behaviour at public places in Biratnagar.

Predictors		Active smoking (n = 325) Yes	No active smoking (n = 400) No	Odds Ratio (95% CI)	P-value
**‘No smoking’ notice**	Present	26 (22.2%)	91 (77.8%)	Ref	<0.001
	Absent	299 (49.2%)	309(50.8%)	3.4 (2.3 to 5.39)	
**Cigarette smell**	Present	240(56.7%)	183(43.3%)	3.3(2.4 to 4.6)	<0.001
	Absent	85(28.1%)	217(71.9%)	Ref	
**Cigarette butts/bidi ends**	Present	258(54.9%)	212(45.1%)	3.4(2.5 to 4.8)	<0.001
	Absent	67(26.3%)	188(73.7%)	Ref	
**Smoking aids**	Present	207(53.8%)	178(46.2%)	2.2(1.6 to 3.0)	<0.001
	Absent	118(34.7%)	222(65.3%)	Ref	
**Active smoking by owners/managers/staff**	Present	106(93.0%)	8(7.0%)	23.7(11.3 to 49.6)	<0.001
	Absent	219(35.8%)	392(64.2%)	Ref	
**Type of Public place**	Education and healthcare institutions	6 (5.8%)	97 (94.2%)	Ref	
	Eateries, entertainment, hospitality, shopping venues, transportations and transits	302 (54.4%)	253 (45.6%)	19.3 (8.3 to 44.8)	<0.001
	Government office buildings and workplaces	17 (25.4%)	50 (74.6%)	5.5 (2.0 to 14.8)	<0.001

The odds of active smoking in places without notice appended was 3.4 (2.1 to 5.4) times compared to that of the places with a ‘no smoking’ notice displayed.

The odds of active smoking in the places where we observed the presence of smell, presence of cigarette butts/bidi ends, and presence of smoking aids were 3.4 (2.4 to 4.6), 3.4 (2.5 to 4.8), and 2.2 (1.6 to 2.9) respectively. The places where owners/managers smoked encouraged active smoking by 23.7 (11.3 to 49.6) times compared to places where owners/managers did not.

In comparison to education and health care institutions, there were 19.3 (8.3 to 44.8) times likely chances of active smoking in eateries, entertainment, hospitality, shopping venues, transportations, and transits. Similarly, workplaces (indoor/outdoor) and government buildings were 5.5 (2.0 to 14.8) times likely to have active smoking in comparison to the education and health care institutions.

Among the variables with a p-value <0.1, when checked for collinearity, we found that the ‘no smoking notice at the entrance’, ‘no smoking notice size compliant with the law’, ‘cigarette smell’, ‘cigarette/bidi stubs/butts’ and ‘active smoking by owners/managers/staffs’ were found to be collinear (with VIF >2). Thus, we dropped these from the multivariable analysis. Multiple binary logistic regression was then applied among the non-collinear variables to obtain the adjusted odds ratio and its 95% confidence interval. After adjusting for collinearity, the final model revealed all the variables except the ‘smoking aids (ashtrays, matchboxes, and lighters)’ was found to be statistically significant. The absence of the ‘no smoking’ notice was found to be 2.1 (1.1 to 3.7) and was statistically significant (p <0.05). Among locations, eateries, entertainments, transportations, and transits were found to have an adjusted odds ratio of 19.8 while government office buildings and workplaces were found to have an AOR of 6.2 compared to the education and healthcare institutions (Table 3).

**Table 3:** Multivariate analysis of factors associated with active smoking.

Characteristics		AOR	95% CI for AOR		p-value
			Lower	Upper	
**‘No smoking’ notice**	Present	Reference			0.011
	Absent	2.1	1.2	3.7	
**Smoking aids**	Yes	0.8	0.5	1.1	0.190
	No	Reference			
**Type of public places**	Education and health care institutions	Reference			
	Eateries, entertainment, hospitality, shopping venues, transportations, transits	19.6	8.1	47.3	<0.001
	Government office buildings and workplaces	6.2	2.3	17.0	<0.001

No public places researched in this study had the content of the ‘no smoking’ notice completely adherent to the requirements of the law (not presented in table).

## Discussion

This study assessed the compliance of smoke-free legislation and identified factors associated with active smoking in public places of a metropolitan city in eastern Nepal. This to our knowledge, is the first peer-reviewed literature reporting the compliance of the smoke-free public places legislation in Nepal. Overall, just over half of the public places (56.4%) were compliant with the provisions set by the smoke-free legislation, with the highest compliance at government office buildings (75.0%) and the lowest at eateries, entertainment, hospitality, and shopping venues (26.3%). Smoking at public places was significantly associated with the absence of a ‘no smoking’ notice at the entrance, the presence of active smoking by owners/managers/staff, and the public place being eateries, entertainment, hospitality and shopping venues or transportations/transits. The content of the ‘no smoking’ notice did not fully adhere to the requirements of the law. The notices either discouraged smoking, mentioned that smoking was prohibited or that smoking was injurious to health. The notice did not mention about legal repercussions as provisioned in the law.

The findings of this study need to be interpreted cautiously keeping in mind that the smoke-free legislation was only operationalized 5 years prior to this study in 2014 and the local level implementation in reality, began only 2 years prior to this study, which is after the local governments became fully functional in 2017 when the local elections were successfully completed. Population compliance and enforcement, concerning smoke-free public places in Nepal, requires further policy action if the country’s ambitions of successfully implementing broader tobacco control legislations are to be realized.

A similar overall compliance as our study, was reported in India, in studies to assess the implementation of smoke-free legislation in the initial years after the implementation of the legislation [25]. Survey results from India during 2012–2013, show 51% overall compliance with smoke-free law which is similar to our study. The compliance rate at educational institutions and healthcare facilities were 65% and 62%, respectively. Lower full compliance rates were seen in the restaurants (37%) and other public places (27%) which included the bus stands, railway stations, shopping malls, stadia, and cinema halls [25]. However, more recent studies show improving trends in compliance with smoke-free laws [21, 25, 26] in India, with a much higher (80%) overall compliance in 2018 [21]. India implemented smoke-free public places legislation in 2008 and it took 10 years of compliance to reach 80% in Chandigarh state [21]. The successful improvement in India was attributed to the robust implementation of the law, involvement of multiple stakeholders and establishment of district-level and state-level tobacco implementation and monitoring cells [21]. It is worth noting that following the implementation of the smoke-free legislation, the country went through precarious political instability and a drastic change in the system of governance. During 2015/16, the federal restructuring as well as the elections took precedence above everything else and that could also have led to the poor follow up on ensuring the compliance of the law. With the new federal structure, which ensures governance on three levels, it should be easier to follow longitudinal compliance of the law.

Smoke-free public places legislation came into effect in 2012 after the Tobacco Product (Control and Regulation) Act 2011 was released. This study, the first of its kind reporting on the compliance of the legislation by public places after 7 years of implementation, shows compliance by just over half the public places. The regulations in Nepal provisions the formation of tobacco control committees at the national level by the Tobacco Control Act 2011. The Tobacco Control Regulation 2011 further outlines the formation of committees at the district and local levels. However, the effectiveness of these committees in the implementation of the law and improving tobacco control activities are yet to be found in the literature. A regulatory committee at the provincial level could be a step to create a link between the national and the local level committees for effective implementation of the legislation nationwide.

The Non-Communicable Diseases Risk Factors STEPS Survey Nepal of 2019 [6] showed a 3.7% absolute reduction (from 37.2% to 33.5%) to secondhand smoke exposure among adults at the workplace compared to the STEPS survey of 2013 [27]. A larger absolute reduction of 13.6% (36.1% to 22.5%) was observed for secondhand smoke exposure at home. These improvements in statistics at home and the workplace also point towards improving social norms. In Nepalese culture, where smoking is still considered an accepted social activity, a changing social norm that decrees such behaviour as hazardous is a welcome change.

Evidence from the literature suggests that tobacco control policies that target anti-smoking social norms, may help people to quit smoking [28–30]. One effective strategy included public anti-tobacco information campaigns focusing on the dangers of second-hand smoke aiming to reduce the social acceptability of smoking thus discouraging people to smoke in public places [29]. This is one area that could be further explored in Nepal for promoting anti-smoking social norms to decrease smoking behaviours in public places.

Our study reported the lowest compliance in eateries, entertainment, and shopping venues (26.3%), which can be compared to studies across the world indicating a higher risk of second-hand smoke exposure in venues such as restaurants and bars [31–34]. Active smoking in public places attended by people of all age groups including children can put the health of non-smokers in serious jeopardy. While it can be debated by the owners that banning smoking can cause them to lose patronage, studies conducted in the US have found otherwise. Studies consistently demonstrated that after the implementation of smoke-free public places in restaurants and bars, patrons were likely to stay longer and more likely to come back. The staff were also more inclined to ask any smoking customer to stop or to smoke outside [35, 36]. The scenario is different in LMICs where the will to implement the law at ground level is still considered low. The managers/staff of restaurants are not motivated to discourage the customers to smoke as their livelihood depends on them [37]. In countries with strong collectivistic societies, the cultural desire to maintain harmony amongst people tends to avert confrontation with smokers [38].

Of particular concern were the findings of compliance in health care facilities of 71.9% with active smoking observed in some of the healthcare facilities surveyed. While studies in India showed healthcare facilities to be the public places with the highest overall compliance rates [21, 25, 26], our study showed otherwise. Further implementation work is needed in healthcare facilities, not least as these public places are visited by people with existing risk factors and vulnerability to the harms of second-hand smoking.

In this study we found that the display of a ‘no smoking’ notice was significantly associated with reduced active smoking in a public place. Studies conducted elsewhere including Uganda, Spain, Scotland, and India found similar associations. Smokers were less likely to smoke if there was discernible notice prohibiting smoking [21, 39–41]. The contents of the ‘no smoking’ notice needs further discussion at policy implementation level, as we found that the content of the message in the current notices did not fully adhere to the requirements of the law.

A similar finding to our study was seen in a study in Punjab, where people were more likely to smoke if the owners/managers of the public places themselves smoked [21]. Different measures can be taken to discourage smoking by the owners/managers which ultimately decreases active smoking. In India, a country with robust implementation of laws regarding smoke-free public places, the manager/owner of the institution is held accountable for the instances where the laws are not followed [21]. The managers and owners should be well trained and updated about the laws regarding smoking in public places including the fines and punishment levied in the cases where these laws are breached.

A major bottleneck hampering the monitoring of effective implementation is the difficulty in incorporating tobacco control reporting into regular reporting [10]. We encountered active smoking in all public places, including health institutions. The presence of active smoking in such places portends how smokers are either unaware of the laws against such acts or that they are not worried about the repercussions. Although fines have been stipulated, the due bureaucratic procedures involved in levying a fine are complicated. Hence the perpetrators are released with a warning rather than going through the trouble of the paperwork involved.

The population compliance rate observed in our study shows a gap between policy action and public health gain. Different rates of compliance in different public places may suggest that there could be success factors from one type of public place that could be useful for another type of public place. There could also be a need for different approaches for different places. A paper from India reports different facilitators and barriers to smoke-free public places legislation that exist in different types of public places [42]. Avoiding negative reactions from guests and having anti-smoking notice taken as an offence by guests were barriers reported by hospitality venues while lack of empowerment to enforce the law were reported by schools as barriers [42]. Public awareness of the law and strong support from the government in implementation including fines were reported as facilitators for policy implementation [42], which is also reported in Nigeria [43]. For eateries, the cleanliness of the venue was regarded as a key facilitator for discouraging customers to smoke [42]. Public awareness could either be done using a ‘grace period’ approach, where the public is educated regarding the policy and provisions before strictly implementing fines or by suing a strict approach where the public are fined immediately giving a message that the government is serious about the law [37]. Therefore, a thorough evaluation of the different approaches used for smoke-free public places in different venues may be beneficial in the future. There is a need not just for robust implementation of the law but also to make the public aware of the existence of such policies [37, 42, 43] Mainstream media and social platforms can be utilized to make sure the people know against smoking in public places. By reaching the younger generations earlier through textbooks and mandated classes, we could begin to change the social norm. A combination of both robust policy implementation to enhance compliance and improvement in social norms that deem smoking in public places dangerous would be necessary to significantly reduce secondhand smoke exposure.

### Limitations of the study

The information on active smoking behaviour was collected based on observation at the places during certain hours of the day for a fixed duration of time. So, slight variation might be observed at other periods. The study tool used did not allow for the collection of data regarding subtypes of the public venues, as compliance could be different between venues within the same category of the public place. We were not able to categorize the public places with further characteristics as located in populated areas or dense areas—the metropolitan office was not able to advise on the categorization. If this was possible, it could have helped generate more useful policy messages. Among the public places chosen, we had to exclude some hotels and health facilities as they did not cooperate with the study. The purposive nature of sampling limits the generalizability of the findings. However, we have taken measures to include all public places without discrimination as they were found during the data collection. Although we have covered a large sample size, we are not able to ascertain if we covered enough as a comprehensive list of public places was not available for generating a sampling frame.

## Conclusion

Our study found the compliance of smoke-free laws in a metropolitan city in Nepal to be satisfactory and progressive given the short duration of implementation and the political turmoil the country was going through parallel to the implementation of the smoke-free law. The findings are valuable in serving as a baseline statistic for future studies to assess compliance. Lower compliance at eateries, entertainment, hospitality, shopping venues, transportations, and transports thus, highlighting the need to be further explored in terms of factors that may facilitate better compliance. This could be explored by the government along with the representatives from these sectors to not only make them aware of such legislation but also the consequences–on health as well as stipulated punishment–in the instances, they are not followed. No smoking notice appended at the main entrance was effective in the reduction of smoking in public places and could be promoted. Future research could further probe the investigation in terms of what factors affected the compliance/non-compliance of the legislation in different venues which could be instrumental in informing policy regarding for effective implementation of smoke-free laws.

## Supporting information

S1 Data (XLSX)

S1 File (PDF)
