# Supplementary material for: Compliance with the smoke-free public places legislation in Nepal: A cross-sectional study from Biratnagar Metropolitan City
Source: PLoS One. 2022 Mar 9;17(3):e0264895. doi: 10.1371/journal.pone.0264895 (PMC8906589; doi:10.1371/journal.pone.0264895)
Supplement: S1 File — (PDF) [file pone.0264895.s002.pdf]

OPTION 1: JURISDICTION WHERE SMOKING IS PROHIBITED INDOORS IN ALL LOCATIONS

PART I. LOCATION INFORMATION

1. Name of location / facility

2. Address

3. Total number of buildings at location (only if you will be observing multiple buildings within one location) (E.g. a university).

4. Type of location (choose only one)
- Hospitals
- Schools
- Bars/ Restaurants
- Industry/ Factory
- Public conveyance
- Government office/ Building
- Bus stands/ Airports
- Hotels etc.

5. Date of visit ________ / ________ / __________

6. Data collector name / code

7. Time of entry to location ________________am / pm

8. Time of departure ________________am / pm

9. Photo taken? ☐ YES ☐ NO

10. Result of observation ☐ Finished ☐ Not Finished

11. If observation not finished, reason why: ☐ Data collector not allowed to enter building/location
☐ Building/location out of business
☐ Other

PART II. OBSERVATION INFORMATION

Observation Indoors

100. Name or number of building (if you will be observing multiple buildings within one location)

Yes No

101. Is anyone smoking tobacco products indoors?

Yes No

102. Do you see a designated smoking area indoors? (Choose “YES” if you see a designated area, even if no one is smoking in it.)

Yes No

103. Are there any ashtrays or ashbins visible indoors?

Yes No

104. Are there one or more no smoking signs in the venue/ location?

Yes No

Observation Outdoors (inside location boundary)

This part of the form can be used if the law requires outdoor areas to be smoke-free.

105. Is anyone smoking within X meters from building entrances and windows?

Yes No

106. Is the location required to be smoke-free outdoors?

Yes No

107. Is anyone smoking tobacco products anywhere outdoors at the location? (If there are no outdoor grounds at this location, choose “not applicable” and finish observation.)

Yes No Not Applicable

108. Are there one or more no-smoking signs *on the grounds of the venue/ location*?

Yes No

109. Are there any ashtrays/ashbin/ ashcans visible *outdoors* at the location?

Yes No

OPTION 2: JURISDICTION WHERE INDOOR DESIGNATED SMOKING AREAS ARE PERMITTED IN SOME TYPES OF LOCATIONS

PART I. LOCATION INFORMATION

1. Name of location / facility

2. Address

3. Total number of buildings at location

- Hospitals
- Schools
- Bars/ Restaurants
- Industry/ Factory
- Public conveyance
- Government office/ Building
- Hotels etc.

5. Date of visit ________ / ________ / __________

6. Data collector name / code

7. Time of entry to location _______________am / pm

8. Time of departure _______________am / pm

9. Photo taken? ☐ YES ☐ NO

10. Result of observation ☐ Finished ☐ Not Finished (Go to Q11) .

11. If observation not finished, reason why: ☐ Data collector not allowed to enter building/location
☐ Building/location out of business
☐ Other

PART II. OBSERVATION INFORMATION

Observation Indoors

100. Name or number of building (if you will be observing multiple buildings within one location)

101. Is *this venue type permitted to have a designated smoking area or room indoors?* (If yes, skip to Q106. If no, continue with Q102 through Q105.)

YES NO

102. Is anyone smoking tobacco products ***indoors***?

YES NO

103. Do you see a designated smoking area ***indoors***? *(Choose "YES" if you see a designated area, even if no one is smoking in it.)*

YES NO

104. Are there any ashtrays or ashbins visible ***indoors***?

YES NO

105. Are there one or more no-smoking signs ***in the venue/ location***?

YES NO

Ask, only if the venue type is permitted to have a designated smoking room or area. Otherwise, proceed to 110.

106. Do you see a designated smoking area ***indoors***? *(Choose "YES" if you see a designated area, even if no one is smoking in it.)*

YES NO

107. Is anyone smoking tobacco products ***indoors, other than in a designated smoking area***?

YES NO

108. Are there any ashtrays or ashbins visible ***indoors, other than in a designated smoking area***?

YES NO

109. Are there one or more no-smoking signs ***in the venue/ location***?

YES NO

Observation Outdoors (inside location boundary):

This part of the form can be used if the law requires outdoor areas to be smoke-free.

110. Is anyone smoking within **X meters from building entrances and windows**?

☐ YES ☐ NO

111. Is the location required to be smoke-free outdoors? *(If yes, continue to Q112. If no, finish observation.)*

☐ YES (Continue to Q112) ☐ NO (Finish observation)

112. Is anyone smoking tobacco products anywhere outdoors at the location? *(If there are no outdoor grounds at this location, choose "not applicable" and finish observation.)*

☐ YES ☐ NO ☐ Not Applicable (Finish observation)

113. Are there one or more no-smoking signs ***on the grounds of the venue/ location***?

☐ YES ☐ NO

114. Are there any ashtrays/ashbin/ ashcans visible ***outdoors*** at the location?

☐ YES ☐ NO
